# Multi-Robot Task Scheduling with Ant Colony Optimization in Antarctic Environments

**DOI:** 10.3390/s23020751

**Published:** 2023-01-09

**Authors:** Seokyoung Kim, Heoncheol Lee

**Affiliations:** Department of IT Convergence Engineering, Kumoh National Institute of Technology, Gumi 39177, Republic of Korea

**Keywords:** Antarctic environments, ant colony optimization, multi-robot task scheduling

## Abstract

This paper addresses the problem of multi-robot task scheduling in Antarctic environments. There are various algorithms for multi-robot task scheduling, but there is a risk in robot operation when applied in Antarctic environments. This paper proposes a practical multi-robot scheduling method using ant colony optimization in Antarctic environments. The proposed method was tested in both simulated and real Antarctic environments, and it was analyzed and compared with other existing algorithms. The improved performance of the proposed method was verified by finding more efficiently scheduled multiple paths with lower costs than the other algorithms.

## 1. Introduction

Due to the development of robot technology, robots are working instead of humans in many places. Robots have the advantage of being able to perform precise tasks that humans cannot do, and tasks that are dangerous for humans to do. Based on these advantages, robots are used in many places such as in homes, services, industry, medical applications, and military applications. Robot applications using a single object, such as a cleaning robot, a guide robot, and a process using a robot arm, have been commercialized. However, in the case of a single robot, the limitations are clear, such as low efficiency or unachievable tasks. To overcome this, multi-robots began to be introduced, which showed increased work efficiency and more diverse mission performance. In other words, using multi-robots enables efficient performance of tasks such as fast processing speed and large workload, but this requires advanced technology. This is because as the number of robots increases, the control structure and calculation time increase dramatically. For this reason, research fields for multi-robots such as task allocation, coverage, and scheduling have been created and are being studied steadily.

Among them, scheduling is for the efficient operation of the robot and aims to reduce the driving time of the robot. When there are multiple robots and multiple destinations, each robot is given an appropriate visit order to minimize the robot’s travel distance and reduce driving time. This can be seen as a kind of traveling salesman problem (TSP) [1,2,3,4,5]. The TSP is to find the shortest possible route from a given set of cities, visiting every city exactly once and returning to the starting point. The TSP is NP-Hard, and there are a number of algorithms to solve this problem. Representatively, there are breadth-first search (BFS) and depth-first search (DFS) [6]. These are algorithms for traversing or searching tree or graph data structures, which guarantees the minimum distance, but has the disadvantage of consuming a lot of resources when the path is long and not guaranteeing a problem-solving time. Nearest neighbor [7] is a simple algorithm to repeat visiting the nearest city. This has the advantage of being easy to implement and fast to calculate, but it does not guarantee minimum distance. The genetic algorithm (GA) [8,9,10,11], one of the heuristic algorithms, guarantees distance and time according to the setting of the user parameter. If the user parameter is properly set, the distance can be calculated with a reasonable calculation time. Ant colony optimization (ACO) [12,13,14,15,16,17,18,19,20] is also a heuristic algorithm that was conceived in the way ants return home in search of food. Various approaches have been taken to solve the TSP using ACO, which also made it possible to obtain distances with reasonable computational time. When the TSP is applied to multi-robots, it is called the multiple traveling salesman problem (MTSP) [21,22,23,24,25,26,27,28]. The MTSP is an optimization problem in which multiple salesmen visit all destinations with minimal distance. Many approaches have been taken to solve the MTSP based on the above algorithms.

**Figure 1:**
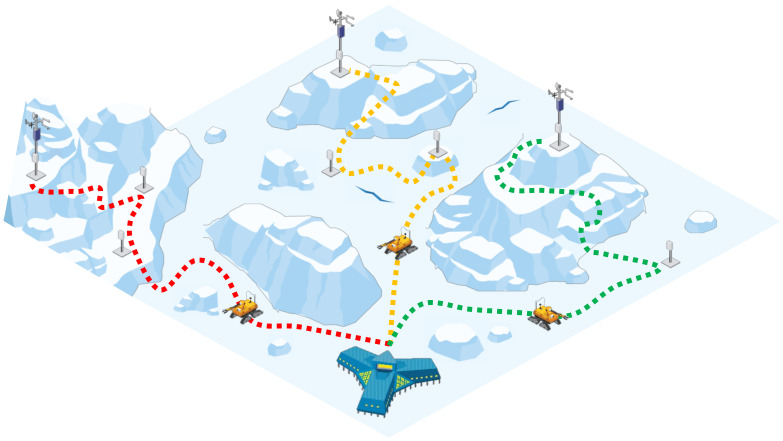
The concept of multi-robot scheduling in Antarctic environments.

However, these TSP solutions will become more difficult to implement in extreme environments. The extreme environment examined here is Antarctica, a place with a large area of about 14,000,000 km^2^, very low temperatures, and various adverse conditions including snow, ice, and crevasses. Antarctica is the southernmost continent of the Earth, and is an attractive unexplored region with enormous scientific value, fishery resources, and energy resources to cope with the Earth’s climate change problems. To discover this value of Antarctica, 47 countries have joined the Antarctic Treaty and are fiercely competing for Antarctic research. Many scientists are trying to study Antarctica, but in extreme weather, crevasses make it difficult for humans to explore the polar regions. To overcome this, scientists have begun to research and introduce unmanned autonomous driving robots. The operation of robots in general environments such as roads and indoors is relatively free from the aforementioned constraints. The general environment does not make robot operation difficult because the floor is relatively flat and not slippery, and there are few fatal obstacles such as crevasses. However, in Antarctic environments, sloping areas such as hills and mountains, slippery floors caused by snow or ice, and crevasses should be considered. The Cold Regions Research and Engineering Lab (CRREL) in the United States has developed ‘Cool Robot’ [29] and ‘Yeti’ [30], autonomous vehicles that can be operated in polar environments, and they are used to collect research data in extreme areas such as Antarctica. However, they show disadvantages in environments such as snow and ice. In addition, the average speed of the robot is about 0.4 to 1.5 m/s, which is slow, and it is somewhat disadvantageous in time to explore the vast area of Antarctica. Therefore, the need for efficient exploration work using multi-robots rather than single robots has emerged. Figure 1 describes the concept of multi-robot scheduling.

In this paper, we propose a practical multi-robot scheduling method in Antarctic environments. This allows multi-robots to visit all nodes with the shortest distance. This can be seen as a kind of MTSP problem-solving, but considering the specificity of Antarctica, the process of returning to the starting point after visiting all nodes was omitted. In addition, stable driving can be realized by avoiding sharp slopes by reflecting Antarctic altitude information in scheduling. It can also produce better results with reasonable computational time. The contributions of this paper are as follows.

- To the best of our knowledge, this is the first approach which solves the multi-robot task scheduling problem in Antarctic environments.
- The performance of the multi-robot task scheduling result was tested and evaluated in both simulated and real Antarctic environments.
- The scheduled paths by the proposed method can improve the efficiency of operating multiple robots by considering the characteristics of robot movement in Antarctic environments.

The remainder of this paper is organized as follows. Section 2 describes constraints in the Antarctic environments and the necessity for scheduling including constraints, as well as the definition of MTSP and problems of applying existing algorithms to Antarctic environments. Section 3 describes the cost function and the structure of ACO used for multi-robot scheduling. Section 4 shows the experimental results and the comparison with other methods. Finally, Section 5 is the conclusion.

## 2. Problem Description

This paper addresses the problem of multi-robot scheduling in Antarctic environments. First, we define the specificity of the Antarctic environment and its problems. It addresses problems that can be caused by extremely low temperatures, snow and ice environments, and altitudes. A practical scheduling method for overcoming these problems is described later. This can be seen as a kind of MTSP, but considering the characteristics of Antarctic environments, it is assumed that the multi-robot does not return to the starting point after visiting all nodes.

### 2.1. Antarctic Environments

Antarctica is one of the coldest regions on Earth, covering an area of about 14,000,000 km^2^, of which 98% is made up of snow and ice. In all regions, the temperature does not exceed 0 °C, and the lowest temperature is −89.2 °C, which is the coldest area. These conditions make it difficult to operate the robot. For example, when exposed to low temperatures, it causes damage to the battery and is bad for the chassis of the robot. Additionally, the snow and ice floor reduce the robot’s ability to move. For stable driving in snow and on icy terrain, it will be necessary to avoid slopes in consideration of height. The crevasse, a deep crack on the glacial surface, is also one of the obstacles that must be avoided. For stable robot operation, these constraints should be avoided as much as possible. Therefore, scheduling in Antarctic environments needs to reflect elements of the terrain as well as distance in the cost.

### 2.2. Definition of MTSP

In this paper, the definition of MTSP is as follows. The multi-robot scheduling problem is defined as visiting a given a set of nodes $C=\left\{c_{1},\;c_{2},\;c_{3},\ldots \;,c_{N}\right\},\;\mathrm{where}\;n=1,\cdots ,N$ for each robot r, with the shortest distance. Each robot has a number of visits $P=\left\{p_{1},\;p_{2},\;p_{3},\ldots \;,p_{R}\right\}$, where r=1,⋯,R. It is defined as a single depot if there is one starting position and a multiple depot if there are multiple starting positions. In this paper, a single depot is assumed. Each of the R robots located in the single depot must visit one or more nodes and will not return to the starting position. Each robot has a tour $T_{r}$, which is described as follows.

(1) $$T_{r}={\left\{{\overset{ˇ}{c}}_{i}^{r}\right\}}_{i=1,\cdots ,p_{R}}\;\mathrm{where}\;{\overset{ˇ}{c}}_{i}^{r}\in C$$

where ${\overset{ˇ}{c}}_{i}^{r}$ is the node visited by the robot r and $p_{r}$ is the total number of nodes that the robot r will visit, calculated as follows.

(2) $$\sum_{r=1}^{R}p_{r}=N$$

In the tour $T_{r}$, when the distance between the nodes ${\overset{ˇ}{c}}_{i}^{r}$ and ${\overset{ˇ}{c}}_{i+1}^{r}$ is $d_{i}^{r}$, the total tour distance for the tour $T_{r}$, is as follows.

(3) $$D\left(T_{r}\right)=\sum_{i=1}^{p_{r}-1}d_{i}^{r}$$

$T_{r,min}$, the tour with the minimum total travel distance, is defined as follows.

(4) $$T_{r,min}=\underset{T_{r}}{\mathrm{argmin}}\left(D\left(T_{r}\right)\right)$$

Then, the goal is to obtain a set of $T_{r,min}$ for R robots.

(5) $$T_{min}=\left\{T_{1,min},T_{2,min},\cdots ,T_{R,min}\right\}$$

To minimize the distance, it is required to set the number of tour nodes $p_{r}$ for each robot r and obtain $T_{min}$ through an appropriate algorithm.

### 2.3. The Problem of Applying the Existing Scheduling Algorithm to Antarctic Environments

Various algorithms have been studied to solve the multi-robot scheduling problem. Among them, the nearest neighbor algorithm is a simple algorithm, summarized as follows.

(1) Select a starting point for any city and register it as a visiting node.
(2) Move to the unvisited node with the lowest cost and register it as the visited node.
(3) Repeat Step 2 if there is a city that was not visited.

It is simple and effective. However, due to the greedy nature of the NN algorithm, it only seeks immediate benefits. Thus, it misses the opportunity to make long-term gains. This leads to the creation of a bad path. When scheduled based on the cost reflecting not only the distance but also the topographical elements, these characteristics will be revealed as disadvantages.

ACO is also one of the algorithms for solving the multi-robot scheduling problem. ACO is an algorithm that solves problems by exploring artificial ants. Ants have a rule that they prefer places with a low cost and high pheromones, which is summarized as follows.

(1) Explore ants.

An ant selects a node by the probability p, which is proportional to the amount of pheromones and the cost.

(2) When the ants finish their search, they leave pheromones in the path of the ant that has the lowest cost.
(3) Repeat as iteration.
(4) After that, the ant that moved to the lowest cost becomes a solution.

In ACO, pheromones as well as cost are additionally considered. Moreover, probabilistic node exploration allows ants to explore various paths, which gives them an opportunity to choose better nodes in the long term. This eventually makes it possible to find a better path.

ACO can easily control the cost function, so it is easy to evaluate factors other than distance. It is also immediate and intuitive because it reflects the cost each time when visiting the nodes one by one.

## 3. Proposed Method

### 3.1. Overview

**Figure 2:**
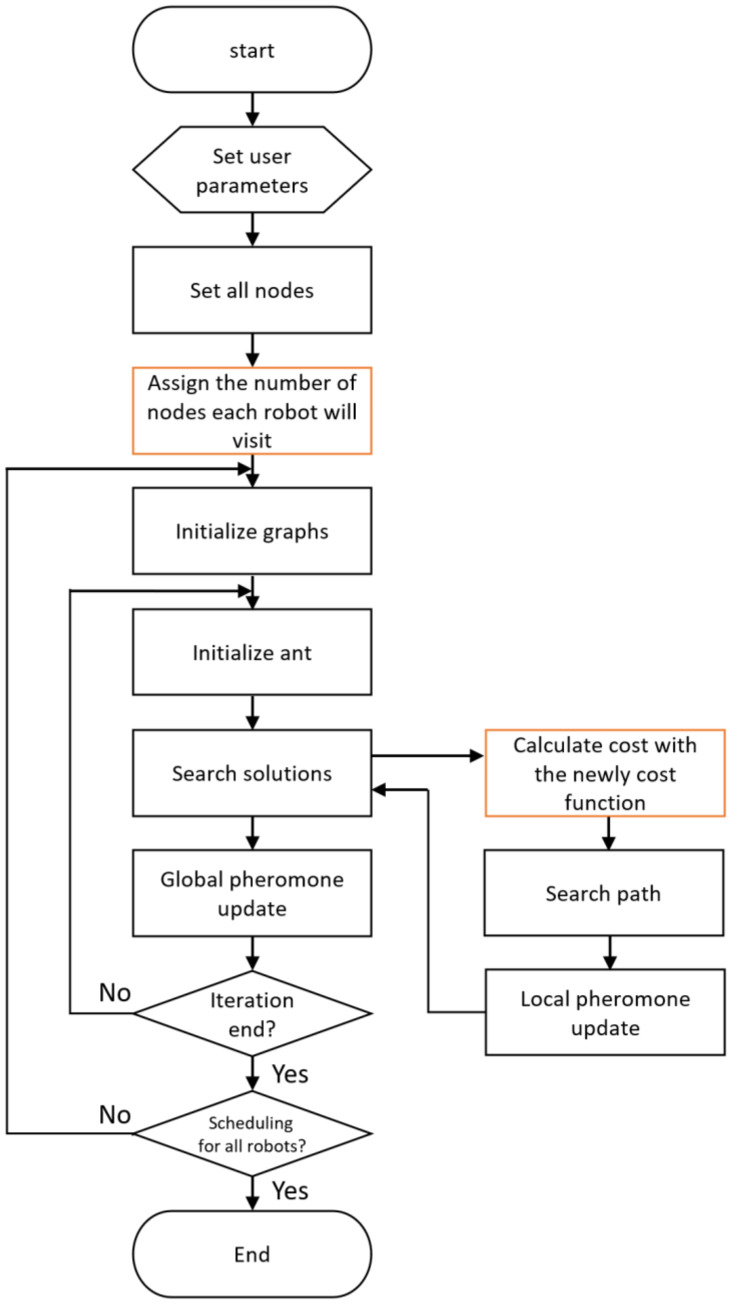
The flowchart of the proposed method.

Figure 2 is a flowchart of the proposed algorithm. This algorithm is based on ACO, but two main features are added. First, for multi-robot scheduling, the number of nodes each robot will visit is set. This is determined by the user or automatically divided by the number of robots. Then, paths for each robot, an MTSP solution, is generated for multi-robots using ACO. After paths for multi-robots are created using the proposed method, each robot moves according to its path. In this paper, a new cost function for ACO is proposed to properly reflect the characteristics of Antarctic environments.

### 3.2. Cost Function

Only the distance between nodes was considered for cost function used in the existing ACO. This is difficult to reflect Antarctic environments. The proposed cost function contains elevation information. In the existing cost function, the distance between nodes p and q is calculated as the Euclidean distance. However, this is a straight distance, which becomes inaccurate if altitude information is added. It is also difficult to measure the exact distance between nodes p and q including altitude information. This was overcome by obtaining an approximate distance value by sampling between nodes. A method of obtaining the distance between nodes p and q including the altitude value is as follows.

The node p and the node q are sampled k times, and the distance obtained by dividing $d_{p,q}$ by k is $d_{k}$, where $d_{p,q}$ is the distance between nodes p and q. The altitude value $H_{p,q}$ sampled between node p and node q is as follows.

(6) $$H_{p,q}=\left(h_{1},h_{2},h_{3,}\cdots \;,h_{k-1},h_{k}\right)$$

where $h_{k}$ is the height of the kth sampled point. The difference $l_{i}$ of the sampled height value is as follows.

(7) $$l_{i}=h_{i+1}-h_{i}$$

The elevation distance $d_{p,q}^{\prime }$ for reflecting the altitude information between nodes *p* and *q* is defined as follows.

(8) $$d_{p,q}^{\prime }=\sum_{i=1}^{k-1}\sqrt{d_{k}{}^{2}+l_{i}{}^{2}}$$

**Figure 3:**
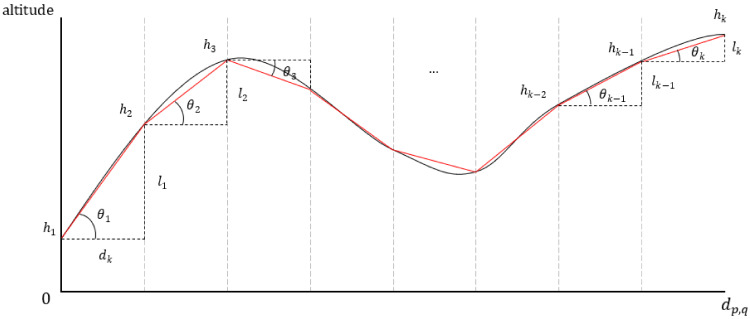
Example of calculating the proposed cost function between nodes p and q.

The value $\Theta_{p,q}$ for reflecting the altitude information between node p and node q is as follows.

(9) $$\Theta_{p,q}=\sum_{i=1}^{k-1}\theta_{i}$$

where $\theta_{i}$ is the angle between the straight line between $h_{i}$ and $h_{i+1}$ and the straight line parallel to the x-axis. Finally, the proposed cost function $c_{p,q}$ is as follows.

(10) $$c_{p,q}=Ad_{p,q}^{\prime }+B\Theta_{p,q}$$

where A and B are weights, which can be arbitrarily determined by the user. $d_{p,q}^{\prime }$ is the distance of the city time, and $\Theta_{p,q}$ is the altitude value of the city time. Figure 3 shows that the cost function is calculated based on the altitude information included at the edge between nodes, where the curve is height information and the straight red line is a straight line connecting points sampled at regular intervals; the sum of the lengths of the straight red line is $d_{p,q}^{\prime }$, and the sum of the angles is $\Theta_{p,q}$.

### 3.3. Ant Colony Optimization

There are various types of ACOs; among them, Ant Colony System (ACS) [13] was used. ACS is an improved algorithm by adding several processes to the existing Ant System (AS) [14,15,16]. Based on this, multi-robot scheduling using cost function adapted to Antarctic environments was implemented.

First, it is the random-proportional rule that ant *k* visits from node p to node q.

(11) $$\omega_{pq}^{k}=\left\{\begin{array}{cc} \frac{\tau_{pq}{}^{\alpha }\eta_{pq}{}^{\beta }}{\sum_{l\notin V_{k}}\tau_{pg}{}^{\alpha }\eta_{pg}{}^{\beta }}, & if\;g\notin V_{k}\\ 0, & otherwise \end{array}\right.$$

where τ is the pheromones and η is the importance of the edge, which is the inverse of the cost. The cost is the value obtained using Equation (10). $V_{k}$ is the set of nodes visited by ant k. α is a parameter that determines the importance of pheromones, and β is a parameter that determines the importance of edge cost. Here, the state transition rule of ACS is applied as follows.

(12) $$s=\left\{\begin{array}{cc} argmax_{q\notin V_{k}}\left\{\tau_{pq}{}^{\alpha }\eta_{pq}{}^{\beta }\right\}, & if\;z<z_{0}\\ S, & otherwise \end{array}\right.$$

where S is a random variable determined by Equation (11). It is a rule about ants choosing a pheromone-rich path. If the random number $z\;\left(0\leq z\leq 1\right)$ is less than $z_{0}$, ants choose the path where the pheromone level is high, and if not, it follows the random-proportional rule of the AS. This prevents falling into the local optimal solution. Random-proportional rules and the state transition rules are applied, and local parent update is performed according to Equation (13) whenever visiting a node.

(13) $$\tau_{pq}=\left(1-\varphi \right)\tau_{pq}+\varphi \tau_{0}$$

where 0≤φ≤1 is a pheromone decay parameter. $\tau_{0}$ is a initial value of pheromone; it is usually $\tau_{0}=1/nc_{nn}$. c is the number of cities, and $c_{nn}$ is the cost calculated by the nearest neighbor. This allows all ants to be affected by pheromones in real time and avoid local optimums.

When all ants generate a tour, global pheromone update is performed through Equations (14) and (15).

(14) $$\tau_{pq}=\left(1-\rho \right)\tau_{pq}+\Delta \tau_{pq}^{best}$$

(15) $$\Delta \tau_{pq}^{best}=\left\{\begin{array}{cc} 1/c_{best}, & if\;best\;ant\;travels\;on\;node\;p,q\\ 0, & otherwise \end{array}\right.$$

where $c_{best}$ is the best ant’s tour. This increases the probability of exploring a better path in the next iteration by accumulating pheromones along the tour of the best solution.

The proposed method appropriately divided the number of nodes so that multi-robots can perform TSP. Although the user may determine the number of nodes to be visited by the robot, it is basically implemented by dividing the number of nodes by the number of robots. Algorithm 1 is a pseudo-code for the proposed method.
**Algorithm 1** Multi-robot scheduling algorithm in Antarctic Environments1:Initialize the multi robot’s tour *T*2:Set number of nodes that the robot will visit *N* and iteration *I*3:**for** *i* ← *N* **do**4: **for**
*j*
←
*I*
**do**:5:  **for** each ant **do**6:   Build a solution according to the number of nodes *i*7:   Update local pheromone8:    
**end for**
9:  Update global pheromone10: 
**end for**
11: Append best ant’s tour to *T*12:**end for**13:**return** Multi robot’s tour *T*

## 4. Results

### 4.1. Results in Simulation Environments

**Figure 4:**
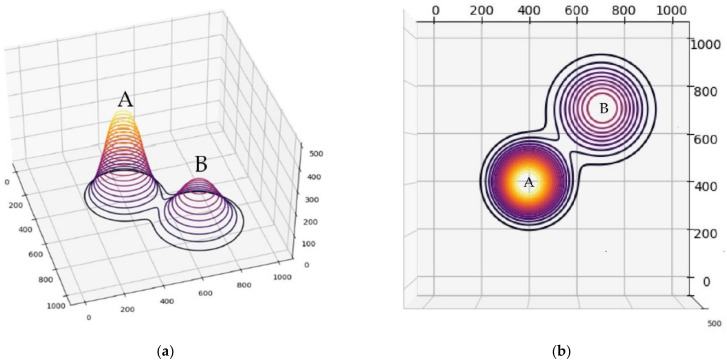
(**a**) The side view of the simulation environment. (**b**) The vertical view of the simulation environment. A and B are the hills of the simulation environment.

Simulations were performed to compare the proposed method with NN and GA. They show a specific performance difference by comparing the elevation distance. The elevation distance uses the value according to Equation (8) according to the x, y, and z coordinates of the node and the edge. The simulations were performed in Python 3.9.7 and the results were visualized using matplotlib. The environment within the simulation is a virtual 3D space, which is 1000 × 1000 × 500 pixels. In order to realize the height in the virtual space, virtual hills A and B according to the normal distribution were generated using the probability density function. The normal distribution function value of hill A is as follows: σ=75, μ=20. The height is 400. The normal distribution function value of hill B is as follows: σ=100, μ=15. The height is 200. The location of the nodes was randomly set, and simulations were performed on 20, 30, and 40 nodes. Figure 4 shows a virtual space, where (a) is the space viewed from the side, and (b) is the space viewed vertically. 

**Figure 5:**
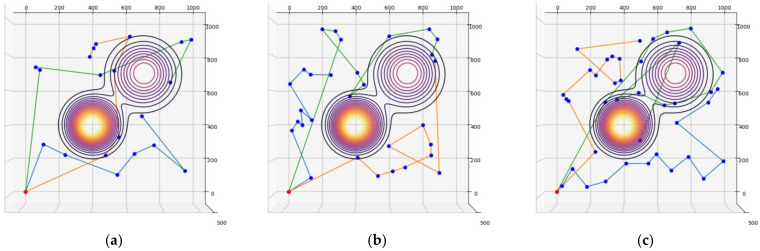
Simulation results of the nearest neighbor in virtual space for: (**a**) 20 nodes, (**b**) 30 nodes, (**c**) 40 nodes. The red dot is the starting point and the blue dots are the nodes to visit. Each color line is the path of each robot.

**Figure 6:**
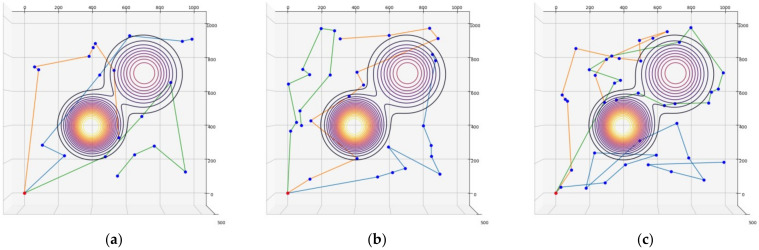
Simulation results of the genetic algorithm in virtual space for: (**a**) 20 nodes, (**b**) 30 nodes, (**c**) 40 nodes.

**Figure 7:**
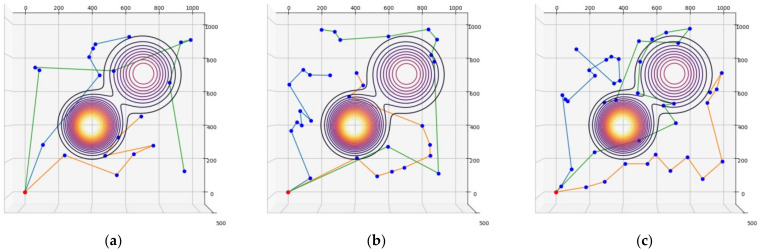
Simulation results of the proposed method in virtual space for: (**a**) 20 nodes, (**b**) 30 nodes, (**c**) 40 nodes.

**Figure 8:**
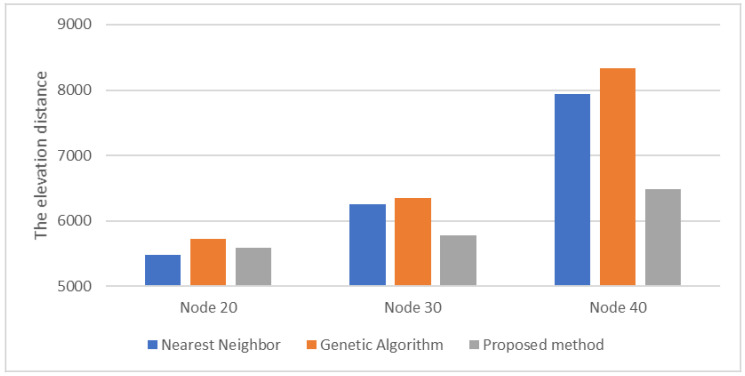
The elevation distance comparison in simulation environments.

For the elevation distance comparison with the proposed method, the nearest neighbor algorithm and the genetic algorithm were performed. The cost functions of the proposed method, NN, and GA were defined according to Equation (10), and the parameter values were as follows: A=10, B=15. The parameter values of GA were as follows: mutation rate=0.05, population=50, generation=300, selection operator was tournament, crossover operator was two-point crossover and elitism was applied. and elitism was applied. The parameter values of ACO in the proposed method were as follows: $ants=40,iteration=20,\alpha =2,\beta =b,\varphi =0.1,\rho =0.05,z_{0}=0.5$, ants is the number of ants, iteration is the number of iterations. Figure 5, Figure 6 and Figure 7 are the results of NN, GA, and the proposed method in the simulation environment, and Table 1 is a comparison table of the elevation distance. Figure 8 is a comparison chart of the elevation distance.

Regarding the results in simulation environments, as shown in Table 1, NN and the proposed method showed similar results for 20 nodes. However, as the number of nodes increased, the proposed method showed a shorter elevation distance. GA showed low performance in all of the results. NN is shorter in computational time, but real time does not need to be guaranteed, so the proposed method is reasonable by generating shorter and more stable paths with less than 10 s.

### 4.2. Results in Real Antarctic Environments

Simulations in Antarctic environments were performed to compare the proposed method with NN and GA. As described above, specific performance differences are presented by comparing the elevation distance.

**Figure 9:**
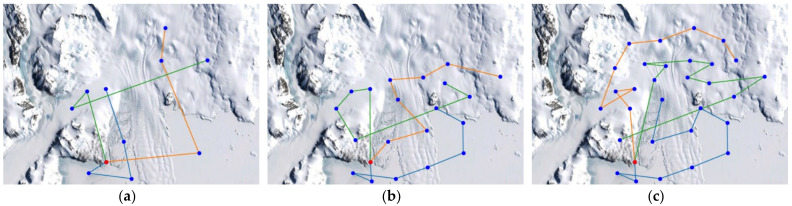
Simulation results of the nearest neighbor in Antarctic environments for: (**a**) 10 nodes, (**b**) 20 nodes, (**c**) 30 nodes. The red dot is the starting point and the blue dots are the nodes to visit. Each color line is the path of each robot.

**Figure 10:**
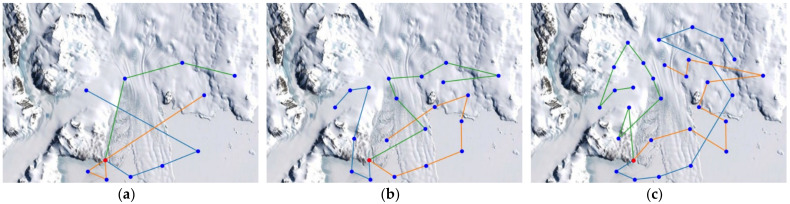
Simulation results of the genetic algorithm in Antarctic environments for: (**a**) 10 nodes, (**b**) 20 nodes, (**c**) 30 nodes.

**Figure 11:**
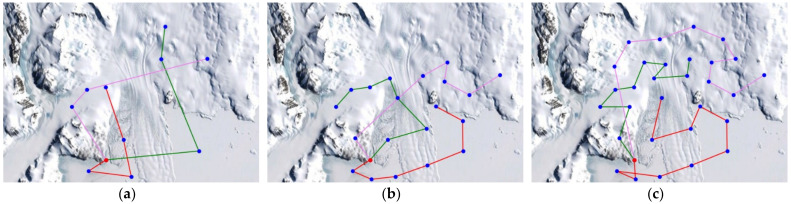
Simulation results of the proposed method in Antarctic environments for: (**a**) 10 nodes, (**b**) 20 nodes, (**c**) 30 nodes.

**Figure 12:**
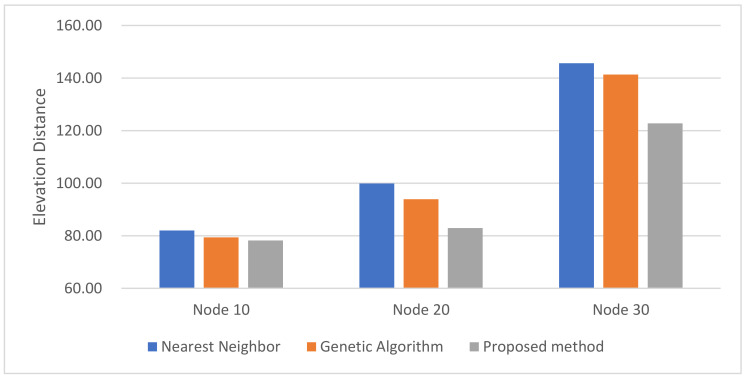
The elevation distance comparison in Antarctic environments.

In the simulation, the Antarctic environment was located at 74°37.4′ S, 164°13.7′ E, and nodes were randomly set nearby. The latitude and longitude values of arbitrary nodes were extracted from Google Earth. The distance between nodes was obtained using the Haversine Formula. The altitude information obtained the altitude values of nodes and edges using the Google Maps API. The altitude values for the edges were sampled 500 times at the same interval. For performance comparison with the proposed method, the nearest neighbor algorithm and the genetic algorithm were performed. The cost functions of the proposed method, NN, and GA were defined according to Equation (10), and the parameter values were as follows: A=3, B=2. The parameter values of GA were as follows: Selection=tournament, Crossover=two−point crossover, mutation rate=0.05, population=50, generation=300, and elitism is applied. The parameter values of ACO in the proposed method were as follows: $ants=40,\;iteration=20,\;\alpha =2,\beta =b,\;\varphi =0.1,\;\rho =0.05,\;z_{0}=0.5$, ants is the number of ants, iteration is the number of iterations. Figure 9, Figure 10 and Figure 11 are the simulation results of NN, GA, and the proposed method in the Antarctic environments, respectively, and Table 2 is a comparison table of the elevation distance. Figure 12 is a comparison chart of the elevation distance.

Regarding the results in Antarctic environments. As shown in Table 2, for all cases, the ACO generated a shorter path, especially when the number of nodes was more than 20, showing better performance. Although NN had a shorter computational time, the proposed method was less than 5 s, which can be considered reasonable if it generates shorter and more stable paths.

## 5. Conclusions

This paper addresses the problem of practical multi-robot task scheduling in Antarctic environments. We analyzed the difficulties of robot operation in Antarctica and present a solution. For stable robot operation, ACO with a novel cost function including altitude information is proposed. The proposed method creates a path that avoids steep slopes, enabling stable robot operation in Antarctic environments consisting of snow and ice. Furthermore, the comparison of the results with the nearest neighbor algorithm shows that the proposed method generates shorter paths, enabling efficient scheduling. However, as mentioned earlier, there are a number of constraints in the Antarctic environment, such as altitude, snow, ice, crevasses, wind speed, and limited communications. In this paper, only a few constraints are considered. Various factors must be considered for more efficient robot operation. In the future, scheduling will be carried out considering various constraints while including altitude information. Stable and efficient robot operation will be possible by further reflecting the factors of Antarctic environments.

## Figures and Tables

**Table 1:** The elevation distance comparison results of the simulation.

Part	Node 20	Node 30	Node 40
Nearest Neighbor	5476.98	6255.67	7943.39
Genetic Algorithm	5729.22	6348.65	8335.68
Proposed Method	5584.36	5784.93	6484.76

**Table 2:** The elevation distance comparison results of real Antarctic environments.

Part	Node 10	Node 20	Node 30
Nearest Neighbor	82.01 km	99.89 km	145.67 km
Genetic Algorithm	79.41 km	93.93 km	141.36 km
Proposed Method	78.17 km	82.95 km	122.78 km

## Data Availability

Not applicable.
